# Triangulation of Hard X-Ray Sources in an X-Class Solar Flare with ASO-S/HXI and Solar Orbiter/STIX

**DOI:** 10.1007/s11207-024-02341-8

**Published:** 2024-08-21

**Authors:** Daniel F. Ryan, Paolo Massa, Andrea F. Battaglia, Ewan C. M. Dickson, Yang Su, Wei Chen, Säm Krucker

**Affiliations:** 1 University of Applied Sciences and Arts Northwestern Switzerland, Bahnhofstrasse 6, Windisch, 5210 Switzerland; 2 Present Address: Mullard Space Science Laboratory, University College London, Dorking, RH5 6NT UK; 3 Institute for Particle Physics and Astrophysics (IPA), Swiss Federal Institute of Technology in Zurich (ETHZ), Wolfgang-Pauli-Strasse 27, 8039 Zurich, Switzerland; 4 Present Address: Istituto ricerche solari Aldo e Cele Daccò (IRSOL), Faculty of informatics, Università della Svizzera italiana, Locarno, Switzerland; 5 Institute of Physics & Kanzelhöhe Observatory, University of Graz, Universitätsplatz 5, 8010 Graz, Austria; 6 Key Laboratory of Dark Matter and Space Astronomy, Purple Mountain Observatory, Chinese Academy of Sciences, Nanjing, 210023 China; 7 School of Astronomy and Space Science, University of Science and Technology of China, Hefei, 230026 China; 8 Space Sciences Lab, UC Berkeley, 7 Gauss Way, Berkeley, CA 94708 USA

**Keywords:** Spectrum, X-ray, Flares, impulsive phase, Flares, spectrum, Instrumentation and data management, Integrated Sun observations

## Abstract

HXI on ASO-S and STIX onboard Solar Orbiter are the first simultaneously operating solar hard X-ray imaging spectrometers. ASO-S’s low Earth orbit and Solar Orbiter’s periodic displacement from the Sun–Earth line enables multi-viewpoint solar hard X-ray spectroscopic imaging analysis for the first time. Here, we demonstrate the potential of this new capability by reporting the first results of 3D triangulation of hard X-ray sources in the SOL2023-12-31T21:55 X5 flare. HXI and STIX observed the flare near the east limb with an observer separation angle of 18°. We triangulated the brightest regions within each source, which enabled us to characterise the large-scale hard X-ray geometry of the flare. The footpoints were found to be in the chromosphere within uncertainty, as expected, while the thermal looptop source was centred at an altitude of 15.1 ± 1 Mm. Given the footpoint separation, this implies a more elongated magnetic-loop structure than predicted by a semi-circular model. These results show the strong diagnostic power of joint HXI and STIX observations for understanding the 3D geometry of solar flares. We conclude by discussing the next steps required to fully exploit their potential.

## Introduction

Hard X-ray emission (HXR; photon energies ≳ 10 keV) is a crucial diagnostic of some of the most energetic processes in solar flares. These include non-thermal electron acceleration and plasma heating to tens of megakelvin, both of which are thought to be among the earliest observable consequences of magnetic reconnection in the solar atmosphere. Our understanding of these processes has been revolutionised (Fletcher et al. 2011; Holman et al. 2011; Kontar et al. 2011) since the launch in 2002 of the Reuven Ramaty High Energy Solar Spectroscopic Imager (RHESSI; Lin et al. 2002). However, further understanding was eventually restricted by limitations including imaging dynamic range and projection effects from the single observing angle.

RHESSI has since been succeeded by two more solar HXR spectroscopic imagers: the Spectrometer/Telescope for Imaging X-rays (STIX; Krucker et al. 2020) onboard Solar Orbiter (Müller et al. 2020), and the Hard X-ray Imager (HXI; Zhang et al. 2019; Su et al. 2019, 2024) onboard the Advanced Space-based Solar Observatory (ASO-S; Gan et al. 2019). Critically, Solar Orbiter operates beyond Earth orbit, while ASO-S maintains a low-Earth orbit. Their launches in 2020 and 2022, respectively, have ushered in the first age of multi-viewpoint solar HXR imaging spectroscopy. The 3D location, morphology and dynamics of HXR sources, revealed by such observations, are integral components of the evolution and energetics of solar flares. For example, rising thermal X-ray sources are often attributed to plasma heating on new flare loops, formed at increasingly higher altitudes by ongoing magnetic reconnection. Meanwhile outward migration of HXR footpoints is thought to be the chromospheric signatures of non-thermally accelerated electrons, conduction fronts, and/or magnetohydrodynamic (MHD) waves propagating downwards along those same newly formed loops. The thermal and non-thermal energies contained within HXR sources are linked to their density and volume, which cannot be accurately estimated from single-viewpoint studies due to projection effects. Multi-viewpoint solar HXR imaging spectroscopy therefore has the potential to shed new light on the initiation, evolution and energetics of solar eruptive events and the high-energy processes that mediate them.

The first multi-viewpoint solar X-ray imaging was performed by Ryan et al. (2024). They combined STIX with the Earth-orbiting Hinode/X-ray Telescope (XRT; Golub et al. 2007) to perform the first 3D reconstruction of an X-ray thermal looptop source. There are several benefits of using XRT in this way, including its high angular resolution and direct-imaging design. These make XRT’s images more straighforward to interpret than the indirect imaging of STIX and HXI. However, while STIX is a spectroscopic imager, XRT uses imaging filters to image plasma at different temperatures. This restricts multi-viewpoint analysis to the thermal X-ray range, and does not allow specific spectral ranges to be extracted from both instruments’ observations. Consequently, combining XRT and STIX for 3D reconstruction is only valid when the sources seen by both instruments are dominated by the same plasma population, which is sometimes difficult to determine. By contrast, HXI is a spectroscopic X-ray imager whose spectral range overlaps significantly with that of STIX. This should make it possible to more reliably, and more often, isolate the same plasma population for 3D reconstruction and other multi-viewpoint X-ray analyses. Moreover, such analyses can be performed as a function of energy, including at higher energies where non-thermal processes dominate. Combining STIX with HXI therefore promises to provide a richer 3D understanding of various X-ray sources in solar flares and the physical mechanisms underpinning them.

In this paper, we take the first steps in multi-viewpoint solar X-ray spectroscopic imaging analysis. We present some of the first joint HXI–STIX observations of an X-class flare, and triangulate the brightest locations in the observed thermal and non-thermal sources. In Section 2, we discuss the HXI and STIX observations and the triangulation methodology. In Section 3, we combine these to derive the 3D locations of the X-ray sources and the properties of the flare loop. In Section 4, we highlight issues that should be addressed by future studies to maximise the potential of solar HXR 3D analyses. Finally, we provide our conclusions in Section 5.

## Observations and Analysis

### Observational Summary

**Figure 1:**
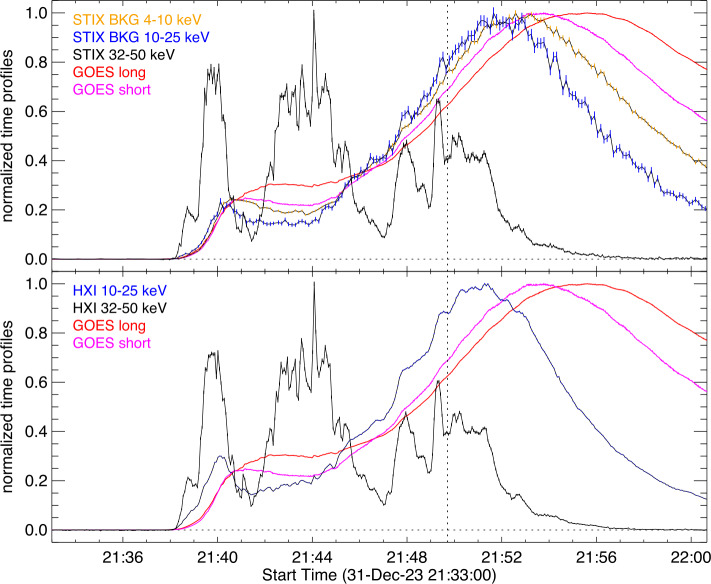
Time evolution of SOL2023-12-31T21:55 in X-rays as seen by GOES, STIX and HXI. The lightcurves are normalised count fluxes with the pre-flare background subtracted. The STIX profiles have been shifted by 16.0 s to account for the different light travel time to Solar Orbiter compared to Earth. Contributions to the HXI lightcurves from particle events during the impulsive phase are negligible. The dashed vertical line gives the time of the AIA images shown in Figure 2.

The X5 flare examined in this study occurred in NOAA active region 13536 on 31 December 2023, and peaked in the GOES 1 – 8 Å channel at 21:55 UT. Figure 1 shows an overview of its soft- and hard X-ray time evolution. As the STIX attenuator moved in around 21:40:23 UT, we used the STIX background detector (Krucker et al. 2020) to derive the time profiles below 25 keV. As its effective area is low, the lightcurves become noisy at the highest energies. The STIX time profile in the non-thermal range (32 – 76 keV) is derived by summing all pixels of the 24 detectors used for imaging. As the non-thermal counts are integrated over many more detectors, it has much smaller error bars. In order to account for the difference in light travel time from the flare to HXI and STIX (16.0 s), we have adjusted the STIX times to coincide with light arrival time at Earth. The HXI time profiles are taken from two detectors with the thick entrance window (D92 and D93) and not covered by imaging grids. To reduce the error bars, all HXR time profiles are summed to a 4 s resolution. The GOES soft X-ray profiles are also shown for context. In order to show all lightcurves on the same scale, they are normalised to their peak flux value.

The impulsive phase of the flare lasts about 16 min with enhanced emission in the non-thermal range (≳ 30 keV). The thermal time profiles below 30 keV show the often-observed behaviour of lower energies peaking later, clearly demonstrating the flare’s multi-thermal nature. The non-thermal emission drastically reduces after the thermal peak, and there are no significant non-thermal bursts during the decay phase.

In order to demonstrate the feasibility of reconstructing the flare geometry by triangulating HXR sources, we investigated a single time interval in the later part of the impulsive phase. The interval is centred around the unsaturated 193 Å image recorded at 21:49:42.6 UT by the Atmospheric Imaging Assembly onboard the Solar Dynamics Observatory (SDO/AIA; Lemen et al. 2012). It was chosen because the HXR geometry at that time exhibited two footpoints, which simplifies co-alignment of the HXR images with the ultraviolet (UV) ribbons observed by AIA (Section 2.2).

### Imaging

HXI and STIX provide spectroscopic imaging in the hard X-ray ranges 10 – 300 keV, and 4 – 150 keV, respectively, with angular resolutions down to $3.1''$ and $7''$. Due to the difficulty of focusing X-rays above ≳ 10 keV, both HXI and STIX employ indirect imaging techniques.^1^ They use pairs of absorbing grids (bi-grids) to modulate the incoming X-ray flux before it is recorded by spectroscopic X-ray detectors (one detector per bi-grid; see Meuris et al. 2012; Zhang et al. 2019). The modulation is sensitive to the position of the X-ray source on the plane of the sky, as well as its angular extent perpendicular to the long axis of the bi-grids. The subcollimators (bi-grid plus detector) have different grid orientations and periods (pitches). They therefore sample different angular Fourier components of the source, more conveniently referred to as (complex) visibilities (Prince et al. 1988; Hurford 2013). The phase and amplitude of each STIX visibility are encoded into a Moiré pattern produced by the X-rays’ transmission through the bi-grid, and recorded by the subcollimator’s pixellated detector (Massa et al. 2023). By contrast, HXI uses non-pixellated detectors, and so employs two subcollimators per visibility to measure the sine and cosine components separately (Zhang et al. 2019). Once measured, the visibilities can be combined to reconstruct an image of the X-ray source using various well-established Fourier-based imaging algorithms (e.g. Piana et al. 2022).

**Figure 2:**
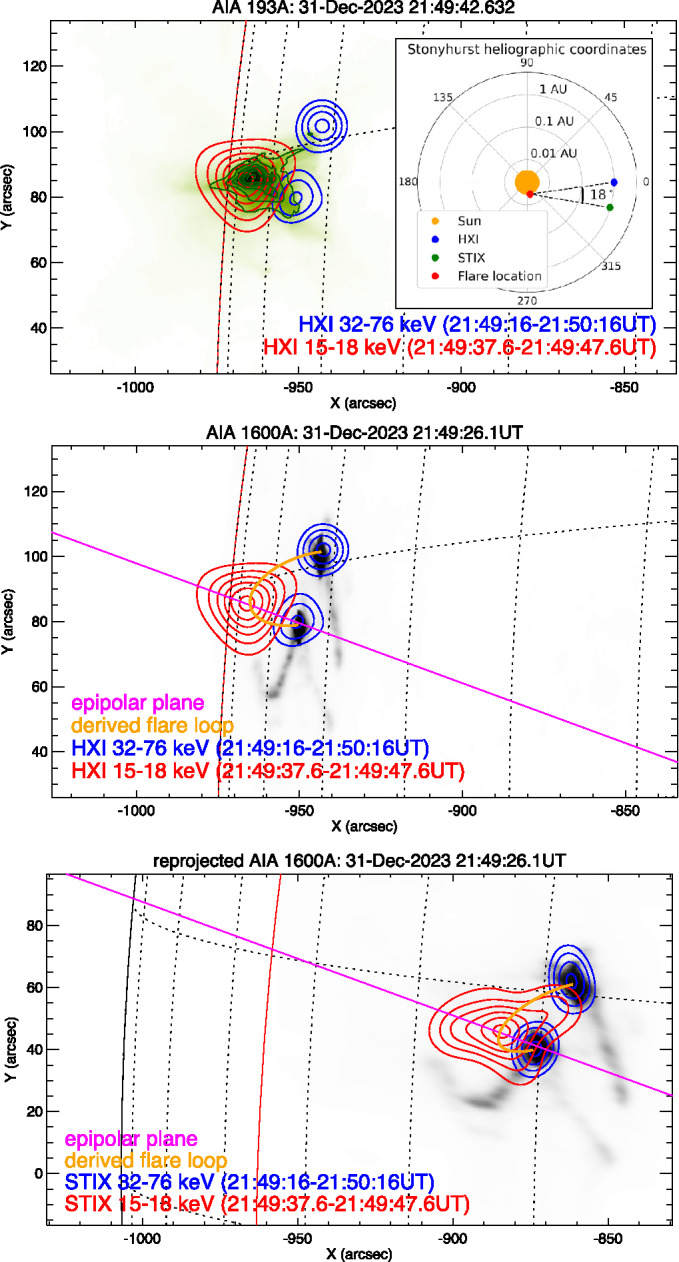
Imaging results in X-rays, EUV and UV around 21:49:42UT. The locations of HXI, STIX, the Sun and the flare are shown in the right inset of the top panel. The distances are plotted on a logarithmic scale to more clearly show the flare location on the Sun. The top and middle panels show thermal (red contours) and non-thermal (blue contours) observed by HXI overlaid on the AIA 193 Å and 1600 Å images, respectively. The bottom panel (see on the next page) shows the STIX HXR sources (thermal, red and non-thermal, blue) overlaid on the AIA 1600 Å image reprojected to the STIX vantage point. The magenta line is the projection of the epipolar plane intersecting Earth, Solar Orbiter and the brightest location of the thermal source (see Section 2.3). It appears as a line because each observer is located on the plane. The epipolar planes through the footpoint sources are not shown. The orange curve is the projection of the 3D loop reconstructed from the source locations. The red curve shows the solar limb as seen from Earth.

Figure 2 shows the 2D image reconstructions of the thermal (15 – 18 keV, red contours, 20 – 95%) and non-thermal (32 – 76 keV, blue contours, 50 – 90%) emission seen by HXI and STIX at 21:49:42 UT.^2^ The thermal and non-thermal images were integrated over intervals of 10 s and 1 min, respectively, centred around this time. This resulted in total imaged thermal counts above background of 116,174 and 19,910 and non-thermal counts above background of 626,282 and 65,441 for HXI and STIX, respectively. All image reconstructions only utilise subcollimators corresponding to angular scales ≥ 14″, because the calibration of both instruments’ finest subcollimators is not yet complete (Massa et al. 2022). In both cases, the Clean image reconstruction algorithm was used, which included a convolution with a Gaussian beam with a full width at half-maximum (FWHM) of $14''$, i.e. the resolution as the finest subcollimators used. HXI background subtraction was performed using a 6-min pre-flare integration centred on 21:33:20 UT. Note that care must be taken in selecting the HXI background due to its high time variability (Liu et al. 2022). By contrast, the STIX background is much more stable in time, and therefore a background estimate was obtained from observations over a non-flaring period on 28 December 2023 between 16:02 to 16:50 UT.

The top and middle panels show the resulting HXI contours overlaid on the AIA 193 Å and 1600 Å images, respectively. The morphology of the thermal HXI reconstruction is consistent with that of the hot (∼ 10 MK) plasma shown by AIA 193 Å. The maximum intensity of the X-ray emission is slightly offset with respect to the core of the extreme ultraviolet (EUV) emission. This may indicate that the higher temperatures to which HXI is more sensitive reside at slightly higher altitudes than those seen by AIA 193 Å. However, as the displacement is less than $2''$, it could instead represent a combination of the alignment and image reconstruction inaccuracies. The next upgrade of the HXI imaging calibration may enable a closer examination of this question. The morphology of the HXI non-thermal footpoints is consistent with the dimension, relative orientation and separation of the UV ribbons (Figure 2, middle panel). The bottom panel shows the AIA 1600 Å map reprojected to Solar Orbiter’s vantage point^3^ and overlaid with the STIX contours. As part of the reprojection, the plate scale of the 1600 Å image has been corrected for the slightly different observer distances. The STIX non-thermal footpoints are also consistent with the reprojected UV flare ribbons, and all STIX sources appear consistent with those seen by HXI, considering the different viewing angles.

**Figure 3:**
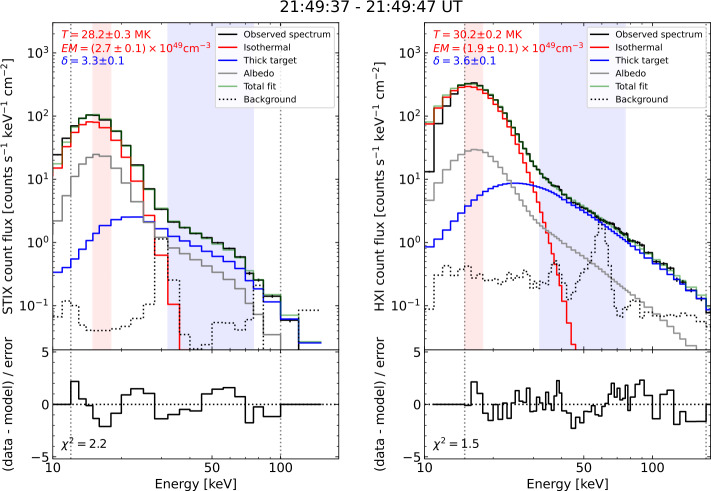
Count spectra from STIX (left) and HXI (right) of the 10 s time interval corresponding to the thermal images in Figure 2. The black histograms show the observed count spectra, while the red (thermal), blue (non-thermal), grey (albedo) and dotted (background) histograms show the components of the best-fit model (green). The lower panels show the residuals between the fit and observations. The vertical dotted lines show the range over which the fit was performed, while the red and blue shaded areas show the spectral ranges corresponding to the thermal and non-thermal images in Figure 2, respectively. The non-thermal spectral indices, $\delta $, are the same within uncertainty, and the peaks of both the count spectra are within the thermal imaging range, where potential differences in the instruments temperature responses are minimised. This suggests that the corresponding HXI and STIX image pairs show the same plasma populations.

Figure 3 shows the count spectra observed during the time interval used for STIX and HXI thermal images. The STIX attenuator was inserted at this time, which drastically reduces the counts at lower energies and hence shifts the peak of the count spectrum to 15 keV. The peak of the HXI count spectrum is at roughly 16 keV, similar to STIX, but is broadened by HXI’s coarser spectral resolution. The spectra were fitted with a model including isothermal, non-thermal thick target and albedo components (red, blue and grey histograms, respectively).^4^ While a detailed discussion of the fitting results is beyond the scope of this paper, it is important to note two things relevant to 3D triangulation. First, both count spectra are dominated by the same model component in the imaged energy ranges (thermal for 15 – 18 keV and non-thermal for 32 – 76 keV). Secondly, the parameters of the components are sufficiently similar for both instruments. Together, this suggests that the brightest subregions of the sources imaged by both instruments’ most likely represent the same 3D location. That said, future studies attempting more detailed 3D reconstructions may require more forensic analyses of the count spectra in the imaged spectral ranges (see Section 4).

A crucial aspect for the analysis in this study is the co-alignment of the STIX and HXI reconstructions. Solar Orbiter’s distance from the Sun (0.95 AU) exceeded the 0.75 AU limit within which the STIX aspect system provides reliable pointing information (Warmuth et al. 2020). To overcome this, the STIX footpoints were co-aligned with the UV ribbons in the reprojected AIA 1600 Å map. This resulted in a shift of $(\Delta x, \Delta y) = (-17'', 29'')$ to the coordinates of the centre of the thermal and non-thermal images, producing a new STIX pointing accuracy of $2''$. The new uncertainty is based on the assumption that the UV ribbons and HXR footpoints were co-spatial, and the fact that shifts beyond $2''$ clearly showed, by eye, a misalignment between the cores of the HXR and UV sources. A similar process was applied to co-align the HXI images with the UV flare ribbons. However, since the HXI pointing information was more reliable at this time, a much smaller shift of $(\Delta x, \Delta y) = (3'', 2'')$ was required. While the nominal pointing accuracy for the HXI observations used at the time of study was < 1″ (Zhang et al. 2019), a more detailed study suggests that calibration of HXI’s aspect system to < 0.3″ should be feasible in the future (Su et al. 2024).

### 3D Triangulation

The SOL2023-12-31T21:55 X-class flare was observed from Heliographic Stonyhurst positions (longitude, latitude and radial distance from Sun centre) 0°, −2.9° and 0.983 AU (HXI, low Earth orbit) and −16.9°, 3.3°and 0.953 AU (STIX, beyond Earth orbit). These positions are shown in the inset in the top panel of Figure 2, along with the corresponding 18° observer separation relative to the flare site. Ryan et al. (2024) showed that the uncertainties associated with full 3D volumetric reconstructions grow rapidly for observer-separation angles less than 70°. (See Appendix B of that paper, specifically Figure B.5.) Therefore, we derived the 3D scalar locations of the brightest subregions of the HXR sources via triangulation. Triangulation does not suffer the same complications as volumetric reconstructions because they depend more on the accuracy with which specific features can be identified from both viewing angles, rather than the extent of their 3D boundaries. The optically thin nature of the X-ray emission and similar viewing angles, and similarities between the instruments’ count spectra (Section 2.2 and Figure 3) suggest that the brightest location in each image should represent the same subregion of the X-ray source. (See Section 4 for further discussion of this issue.)

Performing 3D triangulation requires knowledge of the images’ coordinate frames and the observer locations. The first step is to produce a co-temporal pair of images from STIX and HXI (Figure 2), corrected for pointing misalignments (Section 2.2). Next, an epipolar plane – a 2D plane in 3D space that passes through both observers – must be defined so that it also passes through the feature being triangulated. As both observers sit in the epipolar plane, the plane’s projection onto their images is a line. Therefore, the epipolar plane is determined by simply altering the arbitrary 3rd point, which together with the observer locations defines the plane, until the line of the plane’s projection passes through the feature in both images. This is shown for the case of the thermal source by the magenta lines marked “epipolar plane” in Figure 2. (For a diagram of how epipolar planes relate to the observers and a feature, see Figure 2 of Ryan et al. 2024.) Next, the pixel locations of the feature along the plane are identified in both images. If we then recall that any pixel represents an infinite line of sight stretching away from the observer, it becomes clear that the 3D location of the feature must correspond to the intersection of those lines of sight.^5^ The mathematical details of how to calculate the intersection within the epipolar plane and transform it to 3D real-world coordinates are outlined in Section 4 of Inhester (2006).

## Results

The above methodology (Section 2.3) was applied to the HXI and STIX images (Figure 2) to calculate the 3D coordinates of the brightest locations of the thermal looptop and non-thermal footpoint sources. The coordinate components are shown in Table 1, as well as the sources’ altitudes above the solar surface (1 $\mathrm {R}_{\odot }$). The altitude of the thermal source is similar to estimates at similar stages of previous events (e.g. Gallagher et al. 2002; Milligan, McAteer, and Dennis 2010; Ryan et al. 2024), which gives confidence in the reliability of our triangulation calculations. The 1$\sigma $ uncertainties of the 3D positions^6^ and altitudes were estimated to be ± 2 Mm and ± 1 Mm, respectively. This was determined by assuming that the distribution of pointing uncertainty for each instrument was a Gaussian with a 3$\sigma $ extent equal to the estimated error of the coalignment technique, i.e. $2''$. Random shifts from this pointing distribution were applied to the feature in the STIX and HXI images separately, and the 3D locations were recalculated along with their associated altitudes. The above-quoted uncertainties reflect the standard deviation of the resulting positional distributions.

**Table 1:** 3D locations and altitudes of the brightest regions in the X-ray sources. 3D coordinates are in Heliographic Stonyhurst, while altitudes are defined as above (positive) or below (negative) 1 $\mathrm {R}_{\odot }$ (1 $\mathrm {R}_{\odot }$ = 6.957 × 10^8^ m, in accorrdance with IAU Resolution B3; Mamajek et al. 2015). The uncertainty in the 3D position is ± 2 Mm.

Source	Longitude [deg]	Latitude [deg]	Radius [$\mathrm {R}_{\odot }$]	Altitude [Mm above 1 $\mathrm {R}_{\odot }$]
Thermal looptop	−76.57	4.27	1.022	15.1 ± 1
Northern footpoint	−76.33	5.32	0.998	−1.2 ± 1
Southern footpoint	−77.09	4.00	1.003	1.8 ± 1

This study represents the first determination of the 3D positions of flare footpoints via triangulation of multi-viewpoint HXR spectroscopic imaging observations. Within the above-stated uncertainty of ± 1 Mm, their altitudes above 1 $\mathrm {R}_{\odot }$ place them approximately at the solar surface or slightly above. This is consistent with the footpoints being located in the chromosphere, as expected. This is consistent, within uncertainties, with previous studies that analysed single-viewpoint HXR observations of limb events. They found HXR footpoint altitudes in the ranges 0 – 2.5 Mm (at 30 keV; Brown, Aschwanden, and Kontar 2002; Aschwanden, Brown, and Kontar 2002), 0.8 – 1.7 Mm (Battaglia and Kontar 2011), 0.125 – 0.475 Mm (Martínez Oliveros et al. 2012), and 0.58 – 1.05 Mm (Krucker et al. 2015), including uncertainties.

It may be suggested that the footpoint altitudes found in this study are dictated by the co-alignment process, which requires that the HXR footpoints be aligned with the 1600 Å emission from the flare ribbon in the lower atmosphere. However, we have reason to believe that the derived altitudes are physically representative. In order for the footpoints in each image to correspond to the same point in 3D space, their projections must lie on the same epipolar plane. The co-alignment process was performed without prior knowledge of the 3D position of the sources. Despite this, for each source, an epipolar plane was found that passed through the brightest pixel in both images to within half a pixel. If this were not the case, it would imply an error in the image alignment. However, no adjustments were made post-facto, and the same instrument-specific pointing shifts were used for both the non-thermal and thermal source, which clearly resides at a higher altitude. This strongly suggests that the co-alignments, and hence the derived 3D positions, are representative of the true 3D source locations.

Using the 3D positions in Table 1, we parameterised the flare loop as a semi-ellipse rooted at the footpoints, with its apex in the thermal source (orange curve in Figure 2). The loop properties are shown in Table 2. The loop is significantly elongated into the corona (semi-axis ratio of 0.58, compared to 1 for a semi-circle) and tilted relative to vertical (22.8°). This is generally consistent with higher-resolution EUV images of loops in flaring active regions, which sometimes appear to be substantially inclined. The loop inclination leads to a longer derived loop length (42 Mm) and potentially different apex altitude than might otherwise be inferred from single-viewpoint studies. This may have consequences for the importance of different thermodynamic processes, such as the balance between radiative and conductive cooling (e.g. Cargill, Mariska, and Antiochos 1995; Klimchuk, Patsourakos, and Cargill 2008; Ryan et al. 2013).

**Table 2:** Parameters of the elliptical flare loop defined by the thermal looptop source and footpoints.

Parameter	Value
Loop length	42 Mm
Loop inclination (from surface normal)	22.8°
Footpoint separation (minor axis)	18.8 Mm
Looptop to footpoint midpoint (semi-major axis)	16.1 Mm
Semi-axis ratio (minor/major)	0.58

## Considerations for Future Studies

The results presented in Section 3 clearly demonstrate the potential of stereoscopic HXR imaging spectroscopy for characterising the geometry of high-energy processes in solar flares. In the future, we expect HXI and STIX to jointly observe many more flares, including at greater separation angles better suited to full 3D reconstruction. However, before more detailed studies are performed, a number of issues should be considered.

At present, the uncertainties in the HXR footpoint altitudes do not allow a more detailed consideration of the consequences for models of chromospheric energy deposition in flares than those provided by previous studies. However, triangulation uncertainties would be improved by better knowledge of the absolute source positions in the HXI and STIX images. This could be achieved in the future with improved calibration and a greater separation angle between HXI and STIX. Moreover, triangulation of footpoint sources further from the limb would not be subject to the ambiguities of foreshortening and absorption by optically thick plasma in the lower atmosphere, which had to be considered by those previous studies. Nevertheless, the preferred accuracy for addressing these science questions is of the order 0.1 Mm, which will be challenging to achieve with HXR triangulation.

While indirect imagers, such as HXI and STIX, are well suited to reconstructing large-scale features of X-ray sources, they are much less suited to reconstructing fine details. Care should therefore be given to not over-interpret such fine-scale structure, especially as part of a 3D reconstruction. When comparing HXI and STIX images of the same events, it may be advantageous to only use imaging subcollimators that result in both instruments’ images having a similar spatial resolution, as was done in this study. HXI samples roughly 1.5 times as many visibilites as STIX, including some at finer angular scales ($3.1''$ for HXI compared to $7''$ for STIX). This should enable HXI to reconstruct slightly more morphological detail than STIX. However, when STIX is within ∼ 0.45 AU of the Sun, its angular resolution corresponds to a finer spatial resolution than that of HXI. Therefore, which subcollimators result in the most comparable images will change from event to event.

Multi-viewpoint HXR analysis may also prove useful in disentangling the contribution of albedo in HXR imaging. This topic has proven challenging in the past with previous studies providing inconclusive results (e.g. Battaglia, Kontar, and Hannah 2011). While this topic is beyond the scope of this paper, it should be explored in future studies.

HXI’s spectral 7 keV resolution contrasts starkly with the 1 keV resolution of STIX. When producing HXI images over a spectral range of a few keV, some of the counts will be due to photons outside the selected spectral range. When imaging close to the boundary of a spectral regime – e.g. the transition from thermal to non-thermal – counts associated with sources from the neighbouring spectral range can contaminate the image. This may alter the morphology of the imaged source, or the location of its centroid. The energy range of the thermal images in this paper (15 – 18 keV) are safely distant from this transition, as the non-thermal component only dominates over the thermal above 30 keV.

A similar effect can be induced by the instruments’ different spectral responses, even within the selected spectral range. The spectral response defines the shape of the count spectrum as a function of energy for a given incident photon spectrum. The slope of the count spectrum, combined with the spectral resolution, dictates the weighted average energy of the imaged spectral range. If these are sufficiently different between HXI and STIX, different behaviour may be revealed by their images. For example, if the source is a multi-thermal plasma, the instrument whose image has the lower weighted-average energy will be sensitive to slightly lower temperatures. If these are spatially offset from the higher temperatures, the instruments may show a different source location or morphology. They may also show a different time evolution, because lower temperatures tend to decay slower, as the flare cools first from higher temperatures and then through lower ones. One way of minimising this phenomenon is to image at the peak of the count spectrum, as was done in this study. This was possible because the presence of the attenuator shifted the peak of STIX’s count spectrum to a similar energy to that of HXI (∼ 16 keV). In this scenario, the different spectral responses and resolutions have a smaller impact on the weighted average energies, as their effects are more evenly distributed between lower and higher energies within the spectral range. A more flexible approach, however, may be to image slightly different spectral ranges for the two instruments that nonetheless result in the same weighted-average energy. This would enable a greater exploration of the X-ray source’s 3D structure throughout broader energy and time ranges.

Future 3D analyses that examine the temporal and spectral evolution of HXR sources should be performed in light of a greater understanding of the cross-calibration between HXI and STIX. Such efforts are currently underway by the relevant instrument teams, and should greatly benefit studies like this in the future.

## Summary and Conclusions

We have presented calculations of the 3D locations of the thermal X-ray looptop and non-thermal X-ray footpoint sources during the impulsive phase of the SOL2023-12-31T21:55 X5-class flare. This was achieved by combining observations from ASO-S/HXI and Solar Orbiter/STIX, and represents the first triangulation of solar flare X-ray sources using HXR spectroscopic imaging observations. The footpoints were found to be separated by 18.8 Mm and have altitudes of −1.2 ± 1 Mm and 1.8 ± 1 Mm above one solar radius. The brightest point in the thermal source was found to have an altitude of 15.1 ± 1 Mm. These results are consistent with previous studies that used alternative observations and techniques for estimating the source altitudes. The loop joining the three sources was parameterised as a semi-ellipse and found to be elongated compared to a semi-circle. It had a length of 42 Mm and an inclination of 22.8°.

These results confirm the viability of 3D triangulation of HXR sources using HXI and STIX. More comprehensive 3D volumetric reconstructions were not attempted because the narrow observer separation angle (18°) leads to large uncertainties in the reconstructed volumes of the individual sources. Uncertainties in the triangulation of scalar 3D locations are not so severe as they depend more on the accuracy with which the same features can be identified between images, rather than the 3D source boundaries. We expect HXI and STIX to jointly observe more flares in the future, including many at substantially wider viewing angles, which will enable more comprehensive 3D reconstructions.

In Section 4, we outlined a number of complications that should be considered as part of more detailed studies in the future. These will, in part, be resolved by a better understanding of the calibration and cross-calibration of HXI and STIX. Nonetheless, this paper has demonstrated the current potential of multi-viewpoint HXR observations for scientific exploitation. Ongoing efforts by the HXI and STIX instrument teams to provide the community with better calibrations will only increase that potential. This promises to enable future studies to reconstruct the 3D HXR geometry of flares and eruptions in greater detail than previously possible, and hence elucidate the high-energy processes that underpin them.

## Data Availability

The HXI and STIX data analysed in the current study are available at the ASO-S Data Archive (http://aso-s.pmo.ac.cn/sodc/dataArchive.jsp) and at the Solar Orbiter Archive (https://soar.esac.esa.int/soar/), respectively.

## Footnotes

1 Solar-optimised directly focused hard X-ray observations have been achieved up to ∼ 20 keV by the Focusing Optics X-ray Solar Imager (FOXSI) sounding rocket program (Krucker et al. 2009; Christe et al. 2016). A satellite version of FOXSI would provide directly focused observations up to ∼ 55 keV (Christe et al. 2023; Reid et al. 2023), without the severe count-rate issues intrinsic to NuSTAR solar observing campaigns (Nuclear Spectroscopic Telescope Array; Harrison et al. 2013). Direct focusing has improved capabilities in sensitivity and imaging dynamic range, but remains challenging at higher energies.

2 The HXI and STIX images shown in this paper were reconstructed with the HXI GUI Beta Version 1.40 and the STIX SSWIDL software v0.5.2 (Dickson et al. 2023, Note that a DOI for v0.5.2 is not yet available, so this citation represents v0.5.1).

3 Reprojection was performed assuming the flare ribbons are on the solar surface, and using the sunpy Map class, which leverages astropy reproject (SunPy Community et al. 2020; Mumford et al. 2020; Astropy Collaboration et al. 2022).

4 The spectral fit of both instruments has been performed using the Object Spectral Executive (OSPEX; Schwartz et al. 2002) software available in SSWIDL. For the STIX spectral fit, a spectral response matrix with finer energy binning was used. At present, this feature is not available in the STIX software and it is not yet user-friendly. However, it will be included in a future release.

5 As the lines of sight have been chosen to lie in the same 2D epipolar plane, they must intersect unless they are parallel. They cannot be parallel if the same feature has been identified in both images and the observers’ separation angle is not 0° or 180°.

6 In this analysis, we provide an uncertainty on the 3D location in terms of the radial distance from the derived 3D location. Therefore, we do not provide uncertainties on the coordinates of the location separately.
